# Relationship between Visual Dysfunction and Retinal Changes in Patients with Multiple Sclerosis

**DOI:** 10.1371/journal.pone.0157293

**Published:** 2016-06-28

**Authors:** Maria Satue, Maria Jesus Rodrigo, Sofia Otin, Maria Pilar Bambo, Maria Isabel Fuertes, Jose Ramon Ara, Jesus Martin, Vicente Polo, Jose Manuel Larrosa, Luis Pablo, Elena Garcia-Martin

**Affiliations:** 1 Ophthalmology Department, Miguel Servet University Hospital, Zaragoza, Spain; 2 Aragon Health Research Institute (IACS-IIS Aragon), Zaragoza, Spain; 3 Neurology Department, Miguel Servet University Hospital, Zaragoza, Spain; University of Houston, UNITED STATES

## Abstract

**Aim:**

To evaluate structural changes in the retina and their correlation with visual dysfunction in patients with multiple sclerosis.

**Methods:**

Patients with multiple sclerosis (n = 84) and healthy controls (n = 84) underwent structural evaluation of the retinal nerve fiber layer, and macular and ganglion cell layer thicknesses using Spectral domain optical coherence tomography (SD-OCT). All subjects underwent high and low contrast visual acuity, color vision (using the Farnsworth and L´Anthony desaturated D15 color tests), and contrast sensitivity vision using the Pelli Robson chart and CSV 1000E test.

**Results:**

Macular, retinal nerve fiber layer, and ganglion cell layer thinning was observed in multiple sclerosis patients compared to healthy controls (p<0.05). High- and low-contrast visual acuity and contrast sensitivity vision at four different spatial frequencies were significantly reduced in comparison with healthy subjects (p<0.05). Macular, retinal nerve fiber layer and ganglion cell layer measurements correlated with high and low contrast visual acuity, and contrast sensitivity vision. Contrast sensitivity vision was the functional parameter that most strongly correlated with the structural measurements in multiple sclerosis and was associated with ganglion cell layer measurements. The L´Anthony color vision score (age-corrected color confusion index) was associated with macular measurements.

**Conclusions:**

Patients with multiple sclerosis had visual dysfunction that correlated with structural changes evaluated by SD-OCT. Macular and ganglion cell layer measurements may be good indicators of visual impairment in multiple sclerosis patients.

## Introduction

Optic nerve atrophy and thinning of the peripapillary retinal nerve fiber layer (RNFL) are two typical findings of patients with multiple sclerosis (MS), with or without a history of optic neuritis (ON). Axonal loss is considered to be the main cause of disability in MS.[1–3] Neuronal loss is, however, increasingly recognized as a biomarker that correlates with disability in MS patients.[4–7]

MS is often associated with involvement of the visual pathway that can lead to clinically evident manifestations (such as ON and diplopia) and, more frequently, to subclinical alterations. Several studies have reported a correlation between axonal loss observed in the optic nerve and visual dysfunction in MS.[1,8]. More recently, segmentation analysis of the various retinal layers made possible by new software for digital imaging techniques in ophthalmology have provided a more specific measurement of the retinal ganglion cell layer and the inner plexiform layer complex (GCIPL) and suggest a correlation not only between axonal but also neuronal loss and visual dysfunction in MS patients.[9,10]

In the present study, we assessed macular, RNFL, and GCIPL thicknesses measured by Spectral domain-optical coherence tomography (SD-OCT) using segmentation analysis and evaluated the correlation between structural measurements and visual dysfunction in MS patients.

## Material and Methods

Patients with definite MS and age and sex matched healthy individuals were included in the study. All procedures adhered to the tenets of the Declaration of Helsinki, and the experimental protocol was approved by the Ethics Committee of the Miguel Servet Hospital. All participants provided written informed consent to participate in the study. One eye per subject was randomly selected and included in the study.

The diagnosis of MS was based on standard clinical and neuroimaging criteria and related medical records were carefully evaluated. Information about Expanded Disability Status Scale (EDSS) scores, disease duration, treatments, acute MS attacks, and prior episodes of ON were recorded. The diagnosis of ON was based on clinical findings, which included the presence of decreased visual acuity, relative afferent pupil defect, color vision loss, visual field defect, and a compatible fundus examination. Patients with active ON in the 6 months preceding enrollment in the study or during follow-up were excluded from the study. Participants had no concomitant ocular diseases and no history of glaucoma, retinal pathology, or systemic conditions that could affect the visual system. Eyes with significant refractive errors (>5 D of spherical equivalent refraction or >3 D of astigmatism) were not included in the study.

All subjects underwent a complete neuro-ophthalmic evaluation that included pupillary, anterior segment, and funduscopic examination. We assessed high contrast (HCVA) and low contrast visual acuity (LCVA) using ETDRS and Low-Contrast Sloan Letter Charts, contrast sensitivity vision (CSV) using CVS 1000E and Pelli Robson charts, and color vision (CV) using Farnsworth-Munsell D15 and Lanthony D15 tests. Structural analysis of the retina was performed with SD-OCT using the Cirrus High Definition (HD) OCT (Carl Zeiss Meditec Inc, Dublin, CA), which included 3 applications: macular application (for macular thickness analysis), and RNFL and ganglion cell applications (for individual analysis of these layers). All functional and structural tests were performed during a single visit per patient and measurements were obtained under monocular vision using best correction.

LogMAR visual acuity (VA) was evaluated at three different contrast levels: 100% (HCVA, using ETDRS chart), 2.50%, and 1.25% (LCVA, using Low-Contrast Sloan Letter Charts -Precision Vision, LaSalle, IL-), The percentage indicating the level of contrast, i.e., 100% representing black letters over white background and 1.25% light grey letters over white background. All measurements were obtained under controlled lighting conditions (photopic: mean luminance of 85 cd/m^2^, high mesopic: 5 cd/m^2^, and low mesopic: 3 cd/m^2^).

CSV was evaluated using the Pelli-Robson chart and the CVS 1000E test. The Pelli-Robson chart comprises horizontal lines of capital letters organized into groups of three (triplets) with two triplets per line. Within each triplet, all letters have the same contrast. The contrast decreases from one triplet to the next, even within each line. All patients were evaluated at a distance of 1 meter from the chart, under controlled photopic conditions (85 cd/m^2^). The score corresponding to the last triplet of letters seen by the patient was recorded.

The CSV 1000E instrument is used worldwide for standardized CSV and glare testing. All patients were evaluated at a distance of 2.5 meters from the chart, at four spatial frequencies (3, 6, 12, and 18 cycles per degree [cpd]). The chart comprises 4 rows with 17 circular patches each. The patches present a grating that decreases in contrast moving from left to right across the row. The patient indicates whether the grating appears in the top patch or the bottom patch for each column. Each contrast value for each spatial frequency was transformed into a logarithmic scale according to standardized values.

Color vision was assessed using the Color Vision Recorder program (CVR, Optical Diagnostics, Beusichem, The Netherlands). CVR software is designed for the Windows operating system and analyzes chromatic discrimination by classification of colors. CVR includes several classic color tests. All patients in the study were evaluated using the Farnsworth D15 and L´Anthony D15 protocols (often used to differentiate between subjects with severe loss of color vision and those with milder color defects or normal color vision) and different output parameters, such as the Age-Corrected Color Confusion index (AC CCI, which represents the ratio between the patient’s major radius–largest difference between caps–and the major radius of a perfect arrangement for the subject’s age group), the Confusion angle (Conf angle, which represents the axis of color deficiency), and the Scatter index (S-index, which represents the parallelism of confusion vectors to the personal confusion angle) were recorded.[11,12] All these parameters evaluate the severity of dyschromatopsia. For example, an AC CCI score higher than 1, indicates altered color vision perception; the higher the score in the AC CCI and the S-index, the worse the condition.

Structural measurements of the retina were obtained using the Cirrus OCT device. The same experienced operator performed all scans and did not apply manual correction to the OCT output. We used an internal fixation target because it provides the highest reproducibility and rejected poor quality scans prior to data analysis.[13] We based image quality assessment on the signal strength measurement that combines the signal-to-noise ratio with the uniformity of the signal within a scan (scale 1–10, where 1 is categorized as poor image quality and 10 as excellent). We included images with a score ≥7 for evaluation. The Cirrus OCT macular cube 512 x 128 protocol provides a macular volume measure and retinal thickness values for 9 areas that correspond to the ETDRS. These areas include a central 1 mm circle representing the fovea, and inner and outer rings measuring 3 mm and 6 mm in diameter, respectively. The inner and outer rings are divided into four quadrants each: superior, nasal, inferior, and temporal. The Cirrus OCT optic disc protocol generates 200 x 200 cube images with 200 linear scans enabling analysis of the RNFL of a 6-mm^3^ area around the optic nerve. For each scan series of RNFL measurements, we assessed the average, superior, inferior, temporal, and nasal thickness. The Cirrus segmentation analysis for retinal layers also provides measurements of the GCIPL thickness, evaluating six areas of the macular cube (superior, superonasal, inferonasal, inferior, inferotemporal, and superotemporal sectors). The segmentation analysis also includes measurements of the average and minimum GCIPL. These values are obtained from a set of 360 spokes, where each average represents the mean number of the pixels along that spoke that lies within the measurement annulus. The average and minimum values were selected because they are more sensitive than other retinal measurements for detecting retinal thickness changes.[14]

All data analyses were performed using SPSS software version 20.0 (SPSS Inc., Chicago, IL). The Kolmogorov-Smirnov test was used to assess sample distribution. Differences between evaluations of MS patients and healthy subjects were compared using the Student´s t test. Patients were divided in two groups (history of ON vs no history of ON) and a second analysis to calculate the differences between these two groups was performed using the Student´s t test. The linear correlation between structural and functional parameters was determined using the Pearson correlation coefficient. P values less than 0.05 were considered to indicate statistical significance. To avoid a high false positive rate, the Bonferroni correction for multiple comparisons was calculated and the corrected p values were added to the previously calculated data.

Each eye was considered separately, and one eye from each patient was randomly selected for analysis.

## Results

Eighty-four patients with MS and 84 healthy controls were included in the study. The mean age of the patients with MS was 45.69 (SD = 9.60) and the mean age of the healthy controls was 47.86 (SD = 9.62). Age, sex, and intraocular pressure did not differ significantly between healthy controls and patients with MS (p = 0.140; 0.090 and 0.770 respectively).

All patients had been diagnosed with MS relapse-remitting subtype and were under treatment with interferon 1b (14.3%), glatiramer acetate (4.7%), fingolimod (39.3%), or interferon 1a (10.7%). Only 28.6% of the patients were not under any current treatment. Mean EDSS score was 1.64 (SD: 2.07) and 58% of the patients (n = 49) had a previous ON episode.

### Functional parameters

MS patients showed significant reduction in best-corrected visual acuity at the three contrast levels compared to the controls (0.14±0.67 in patients vs -0.09 ± 0.09 in controls at 100%, p = 0.010; 0.55±0.17 vs 0.42±0.12 at 2.5%, p<0.001; 0.71±0.16 vs 0.55±0.14 at 1.25%, p<0.001). CSV was affected in patients in all four spatial frequencies of the CSV 1000E chart (3, 6, 12, and 18 cpd) when analyzed based on the number of correct localized gratings (p<0.001). The Pelli Robson results revealed a significant reduction in CSV in MS patients (p<0.001). L´Anthony AC-CCI and Conf angle results were also significantly worse in MS patients. The results are shown in Table 1.

**Table 1:** Visual function and structural parameters in healthy controls and subjects with multiple sclerosis.

	CONTROL	MS	P
**FUNCTIONAL EXAMINATION**			
**VISUAL ACUITY**			
*ETDRS 100*	-0.09 (0.09)	0.14 (0.67)	0.010*
*ETDRS 2*.*5*	0.42 (0.12)	0.55 (0.17)	<0.001*
*ETDRS 1*.*25*	0.55 (0.14)	0.71 (0.16)	<0.001*
**CONTRAST SENSITIVITY**			
*Pelli Robson*	1.89 (0.11)	1.74 (0.23)	<0.001*
*CSV 1000 3 cpd*	1.73 (0.19)	1.44 (0.23)	<0.001*
*CSV 1000 6 cpd*	1.98 (0.19)	1.50 (0.31)	<0.001*
*CSV 1000 12 cpd*	1.62 (0.22)	1.00 (0.27)	<0.001*
*CSV 1000 18 cpd*	1.16 (0.21)	0.56 (0.11)	<0.001*
**CHROMATIC VISION**			
*CVR Farnsw AC CCI*	1.11 (0.43)	1.13 (0.24)	0.660
*CVR Farnsw ConfAngle*	56.08 (7.93)	59.10 (8.63)	0.520
*CVR Farnsw S- Index*	1.66 (0.43)	1.74 (0.48)	0.310
*CVR L’Anthony AC CCI*	1.13 (0.36)	1.26 (0.30)	**0.030**
*CVR L’Anthony ConfAngle*	55.49 (4.02)	40.27 (6.49)	**0.040**
*CVR L’Anthony S-Index*	1.79 (0.50)	1.81 (0.41)	0.856
**STRUCTURAL EXAMINATION**			
**MACULAR THICKNESS**			
*Fovea*	257.07 (16.33)	249.01 (16.34)	**0.012**
*Inner superior sector*	325.99 (13.37)	309.86 (22.47)	<0.001*
*Inner nasal sector*	326.35 (13.56)	309.61 (24.02)	<0.001*
*Inner inferior sector*	321.96 (14.00)	304.44 (23.52)	<0.001*
*Inner temporal sector*	311.14 (12.24)	297.67 (21.70)	<0.001*
*Outer superior sector*	283.21 (11.66)	273.44 (19.06)	<0.001*
*Outer nasal sector*	299.80 (14.97)	282.06 (23.60)	<0.001*
*Outer inferior sector*	272.04 (13.02)	262.11 (27.91)	**0.030**
*Outer temporal sector*	264.79 (11.86)	254.67 (14.27)	0.540
*Average*	291.55 (15.70)	273.70 (18.47)	<0.001*
*Volume*	10.12 (0.54)	9.88 (0.66)	<0.001*
**GCIPL THICKNESS**			
*Superior sector*	85.58 (6.67)	75.91 (8.46)	**0.040**
*Superonasal sector*	85.85 (7.46)	75.87 (9.25)	**0.030**
*Inferonasal sector*	84.61 (7.55)	75.26 (9.58)	0.140
*Inferior sector*	83.59 (7.70)	74.30 (9.56)	0.130
*Inferotemporal sector*	84.17 (6.73)	73.91 (13.11)	**0.040**
*Superotemporal sector*	83.87 (6.23)	73.35 (10.70)	**0.010**
*Average GCIPL*	84.68 (6.74)	74.87 (8.54)	0.080
*Min GCIPL*	82.42 (6.50)	70.17 (13.83)	<0.001*
**RNFL THICKNESS**			
*Average*	94.35 (9.62)	84.34 (13.14)	0.010*
*Superior sector*	117.10 (17.20)	107.08 (17.71)	0.550
*Nasal sector*	69.93 (11.79)	67.50 (14.07)	0.230
*Inferior sector*	124.1 (14.81)	107.63 (18.70)	0.010*
*Temporal sector*	64.14 (8.94)	55.5 (18.52)	<0.001*

Mean and standard deviation (SD) of visual function and structural parameters in healthy controls and subjects with multiple sclerosis. The Student T test was performed to compare controls and patients with MS. Results in bold letters indicate statistical significance (p<0.050). Asterisk indicates a significant difference by Student’s t test after Bonferroni correction for multiple tests (p≤0.017 for ETDRS; p≤0.010 for CSV 1000E measurements; p≤0.0083 for Farnsworth and L´Anthony tests; p≤0.0056 for macular thickness values; p≤0.00625 for GCIPL thickness and p≤0.010 for RNFL thickness). Abbreviations: ETDRS, Early Treatment Diabetic Retinopathy Study; cpd, cycles per degree; AC CCI, age-corrected color confusion index; Conf Angle, confusion angle; S-index, scatter index; GCIPL, ganglion cell +inner plexiform layer; RNFL, retinal nerve fiber layer; HD, high definition; MS, multiple sclerosis.

ANOVA was used to calculate differences between controls and patients with and without a previous episode of ON (ON vs no-ON) (S1 Table). The EDSS score was significantly worse in patients without a previous episode of ON (2.43±2.50 in patients without an ON episode vs 1.14 ±1.47 in patients with an ON episode, p<0.001). The post hoc analysis revealed that functional parameters were only different between controls and patients (with and/or without a history of ON); no differences were observed between the two groups of patients (S1 Table).

### Structural parameters

OCT measurements indicated significant differences in almost all macular sectors (except in the central and outer temporal thickness; Table 1). The segmentation analysis revealed reduced GCIPL thickness in MS patients in the inferotemporal (73.9±13.1 μm in patients vs 84.2±6.7 μm in controls; p = 0.04) and superotemporal sectors (73.4±10.7 μm vs 83.9±6.2 μm; p = 0.01). The minimum GCIPL value was significantly reduced (70.2±13.8 μm vs 82.4±6.5 μm; p<0.001). The RNFL was significantly reduced in the average thickness and temporal quadrant in MS patients (Table 1).

The ANOVA analysis revealed a significant difference in all GCIPL measurements (S2 Table). Post hoc analysis of macular measurements revealed significant differences between healthy controls and patients (with and without a previous ON episode) in several sectors; however, differences between the two subgroups of patients were only observed in the foveal and average macular thickness, and macular volume.

Post hoc analysis of GCIPL thickness revealed statistical differences between controls and patients (with and without a previous ON episode) in the superior and superonasal sectors, and in the minimum GCIPL thickness.

### Correlation between functional and structural parameters

**Fig 1:**
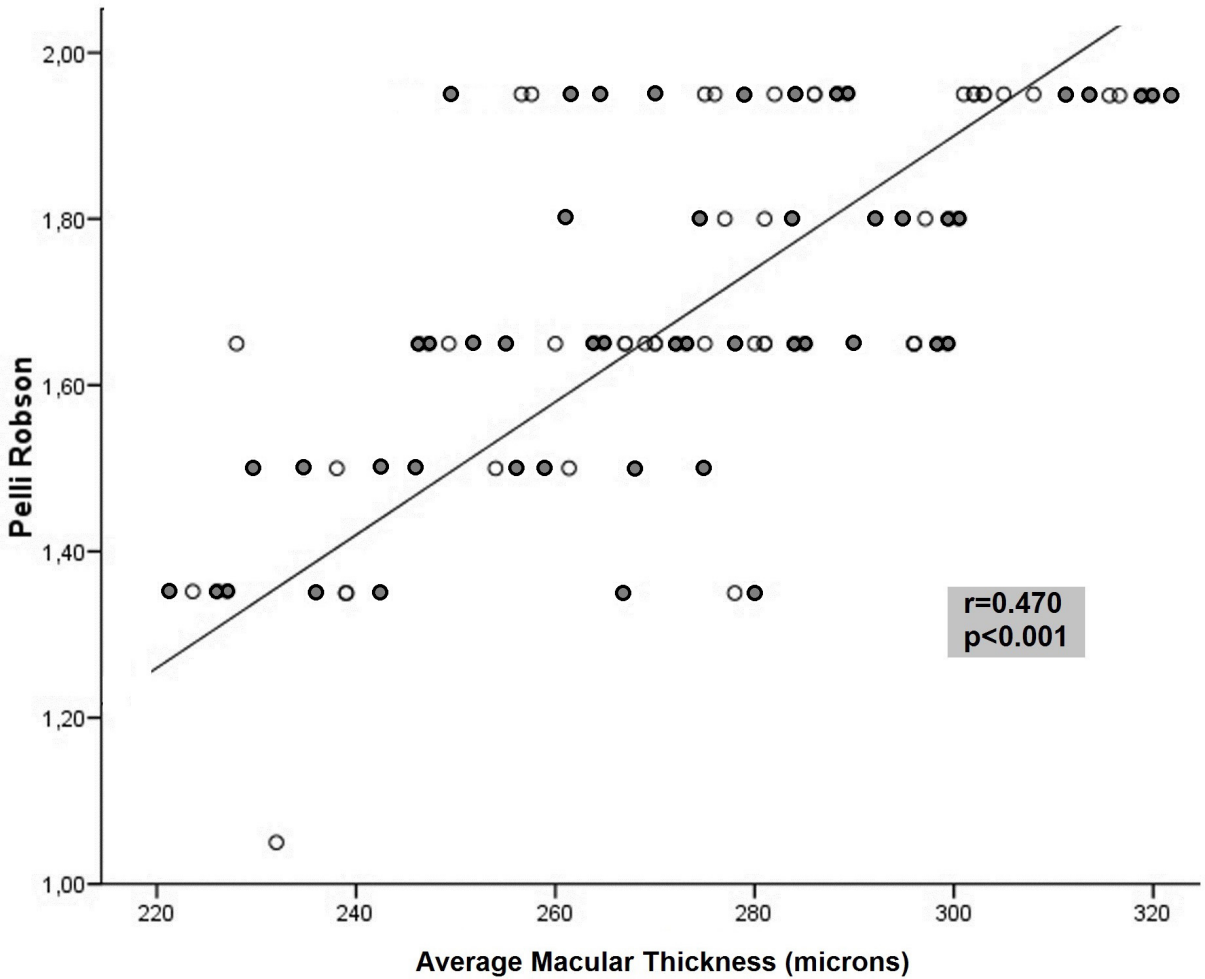
Correlation between the average macular thickness and contrast sensitivity vision measured with the Pelli Robson test in patients with multiple clerosis. Dark symbols represent data from patients with a previous episode of optic neuritis, whereas light symbols represent patients without a previous episode of optic neuritis.

**Fig 2:**
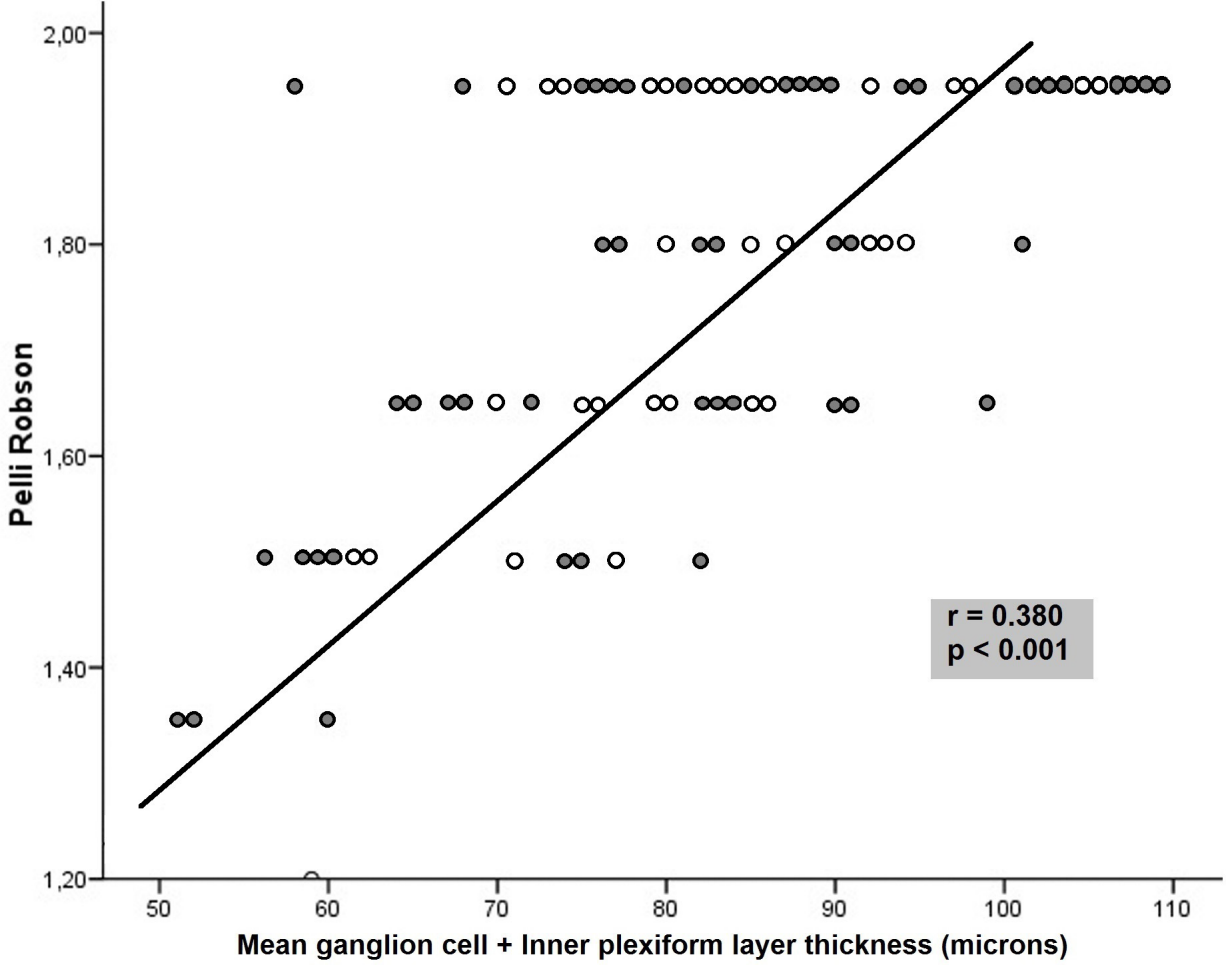
Correlation between the average ganglion cell + inner plexiform layer thickness and contrast sensitivity vision measured with the Pelli Robson test in patients with multiple sclerosis. Dark symbols represent data from patients with a previous episode of optic neuritis, whereas light symbols represent patients without a previous episode of optic neuritis.

CSV was the functional parameter most frequently associated with structural measurements in MS. The Pelli Robson CSV results correlated with GCIPL thickness in all sectors and macular thickness in 8 of 9 sectors, although the association was not strong (r < 0.5) (Figs 1 and 2). The outer temporal (r = 0.41, p<0.001) and average macular thickness (r = 0.47, p<0.001) values had the highest correlations. The Pelli Robson results also correlated with the thickness in different sectors of the RNFL (average, superior, inferior, and temporal sectors, r<0.50, p<0.05). Measurements with the CSV 1000E at different spatial frequencies correlated significantly with most GCIPL measurements (r<0.50). The superotemporal (r = 0.50, p< 0.001) thickness, average GCIPL thickness (r = 0.48, p< 0.001), and minimum GCIPL (r = 0.50, p< 0.001) thickness had the strongest correlations at a spatial frequency of 18 cpd (Table 2).

**Table 2:** Correlation between structural parameters and contrast sensitivity vision in patients with multiple sclerosis.

	CSV 1000E test								Pelli Robson	
STRUCTURAL MEASUREMENTS	*3 cpd*	*P*	*6 cpd*	*p*	*12 cpd*	*p*	*18 cpd*	*p*	*r*	*P*
**MACULAR THICKNESS**										
*Fovea*	0.15	0.080	0.15	0.080	0.12	0.180	0.18	**0.040**	0.12	0.150
*Inner superior sector*	0.32	<0.001*	0.41	<0.001*	0.41	<0.001*	0.42	<0.001*	0.36	<0.001*
*Inner nasal sector*	0.34	<0.001*	0.45	<0.001*	0.41	<0.001*	0.44	<0.001*	0.35	<0.001*
*Inner inferior sector*	0.32	<0.001*	0.42	<0.001*	0.42	<0.001*	0.41	<0.001*	0.35	<0.001*
*Inner temporal sector*	0.30	<0.001*	0.37	<0.001*	0.38	<0.001*	0.41	<0.001*	0.31	<0.001*
*Outer superior sector*	0.30	<0.001*	0.33	<0.001*	0.36	<0.001*	0.37	<0.001*	0.39	<0.001*
*Outer nasal sector*	0.35	<0.001*	0.43	<0.001*	0.38	<0.001*	0.44	<0.001*	0.34	<0.001*
*Outer inferior sector*	0.17	0.050	0.28	0.001*	0.27	0.002*	0.31	<0.001*	0.27	<0.001*
*Outer temporal sector*	0.33	<0.001*	0.30	<0.001*	0.40	<0.001*	0.41	<0.001*	0.41	<0.001*
*Average*	0.29	**0.020**	0.38	0.003*	0.39	0.002*	0.44	0.001*	0.47	<0.001*
*Volume*	0.17	0.180	0.34	**0.006**	0.27	**0.030**	0.30	**0.018**	0.15	0.230
**GCIPL THICKNESS**										
*Superior sector*	0.22	**0.020**	0.37	<0.001*	0.37	<0.001*	0.44	<0.001*	0.40	<0.001*
*Superonasal sector*	0.21	**0.030**	0.38	<0.001*	0.36	<0.001*	0.41	<0.001*	0.38	<0.001*
*Inferonasal sector*	0.18	**0.060**	0.37	<0.001*	0.36	<0.001*	0.41	<0.001*	0.37	<0.001*
*Inferior sector*	0.19	**0.050**	0.41	<0.001*	0.35	<0.001*	0.45	<0.001*	0.38	<0.001*
*Inferotemporal sector*	0.23	**0.020**	0.36	<0.001*	0.34	0.001*	0.42	<0.001*	0.25	<0.001*
*Superotemporal sector*	0.31	0.002*	0.45	<0.001*	0.44	<0.001*	0.50	<0.001*	0.36	<0.001*
*Average GCIPL*	0.25	**0.010**	0.43	<0.001*	0.40	<0.001*	0.48	<0.001*	0.38	<0.001*
*Min GCIPL*	0.27	**0.007**	0.43	<0.001*	0.42	<0.001*	0.50	<0.001*	0.36	<0.001*
**RNFL THICKNESS**										
*Average*	0.18	**0.030**	0.19	**0.020**	0.14	0.090	0.15	0.070	0.33	<0.001*
*Superior sector*	0.15	0.070	0.13	0.110	0.13	0.120	0.16	0.060	0.30	<0.001*
*Nasal sector*	0.02	0.790	0.08	0.350	0.16	0.060	0.15	0.070	0.08	0.310
*Inferior sector*	0.15	0.060	0.16	**0.040**	0.11	0.200	0.09	0.260	0.32	<0.001*
*Temporal sector*	0.24	0.004*	0.34	<0.001*	0.34	<0.001*	0.35	<0.001*	0.24	<0.001*

Correlation between structural parameters (macular, ganglion cell layer and retinal nerve fiber layer thickness) and contrast sensitivity vision (CSV) evaluated with the CSV 1000E and Pelli Robson tests in patients with multiple sclerosis. Data in bold type correspond to statistically significant correlations (p-value <0.05). Asterisk indicates a significant difference after Bonferroni correction for multiple tests (p≤0.0056 for macular thickness values; p≤0.00625 for GCIPL thickness and p≤0.010 for RNFL thickness). Abbreviations: GCIPL, ganglion cell+inner plexiform layer; RNFL, retinal nerve fiber layer; cpd, cycles per degree.

Color vision was also associated with structural parameters: Farnsworth´s AC-CCI was significantly associated with the GCIPL thickness (superior, superonasal, inferonasal, and the average GCIPL thickness), although the association was not strong (r<0.30; Table 3). L´Anthony´s AC-CCI was statistically correlated with different macular sectors (Table 3). Macular average thickness showed the highest correlations with the color vision indexes (Farnsworth´s AC-CCI r = -0.53, p<0.001 [data not shown in tables]; L´Anthony´s AC-CCI r = -0.47, p<0.001). The color vision parameters were not significantly correlated with the RNFL thickness.

**Table 3:** Correlation between structural measurements and color vision in patients with multiple sclerosis.

**FARNSWORTH COLOR TEST**		
GCIPL THICKNESS	AC-CCI	p
*Superior sector*	-0.21	**0.030**
*Superonasal sector*	-0.23	**0.020**
*Inferonasal sector*	-0.23	**0.010**
*Inferior sector*	-0.16	0.100
*Inferotemporal sector*	-0.16	0.100
*Superotemporal sector*	-0.21	**0.030**
*Average GCIPL*	-0.23	**0.020**
*Min GCIPL*	-0.13	0.190
**L´ANTHONY COLOR TEST**		
MACULAR THICKNESS	AC-CCI	p
*Fovea*	-0.01	0.900
*Inner superior sector*	-0.21	**0.020**
*Inner nasal sector*	-0.25	<0.001*
*Inner inferior sector*	-0.22	**0.010**
*Inner temporal sector*	-0.17	0.060
*Outer superior sector*	-0.18	0.050
*Outer nasal sector*	-0.10	0.290
*Outer inferior sector*	-0.05	0.580
*Outer temporal sector*	-0.06	0.530
*Average thickness*	-0.47	<0.001*

Correlation between structural measurements (ganglion cell layer and macular thickness) and color vision evaluated with the Farnsworth and L´Anthony color tests in patients with multiple sclerosis. Correlation data in bold type are statistically significant (p-value <0.05). Asterisk indicates a significant difference after Bonferroni correction for multiple tests (p≤0.00625 for GCIPL thickness and p≤0.0056 for macular thickness values). Abbreviations: GCIPL, ganglion cell+inner plexiform layer; AC-CCI: age-corrected color confusion index.

**Fig 3:**
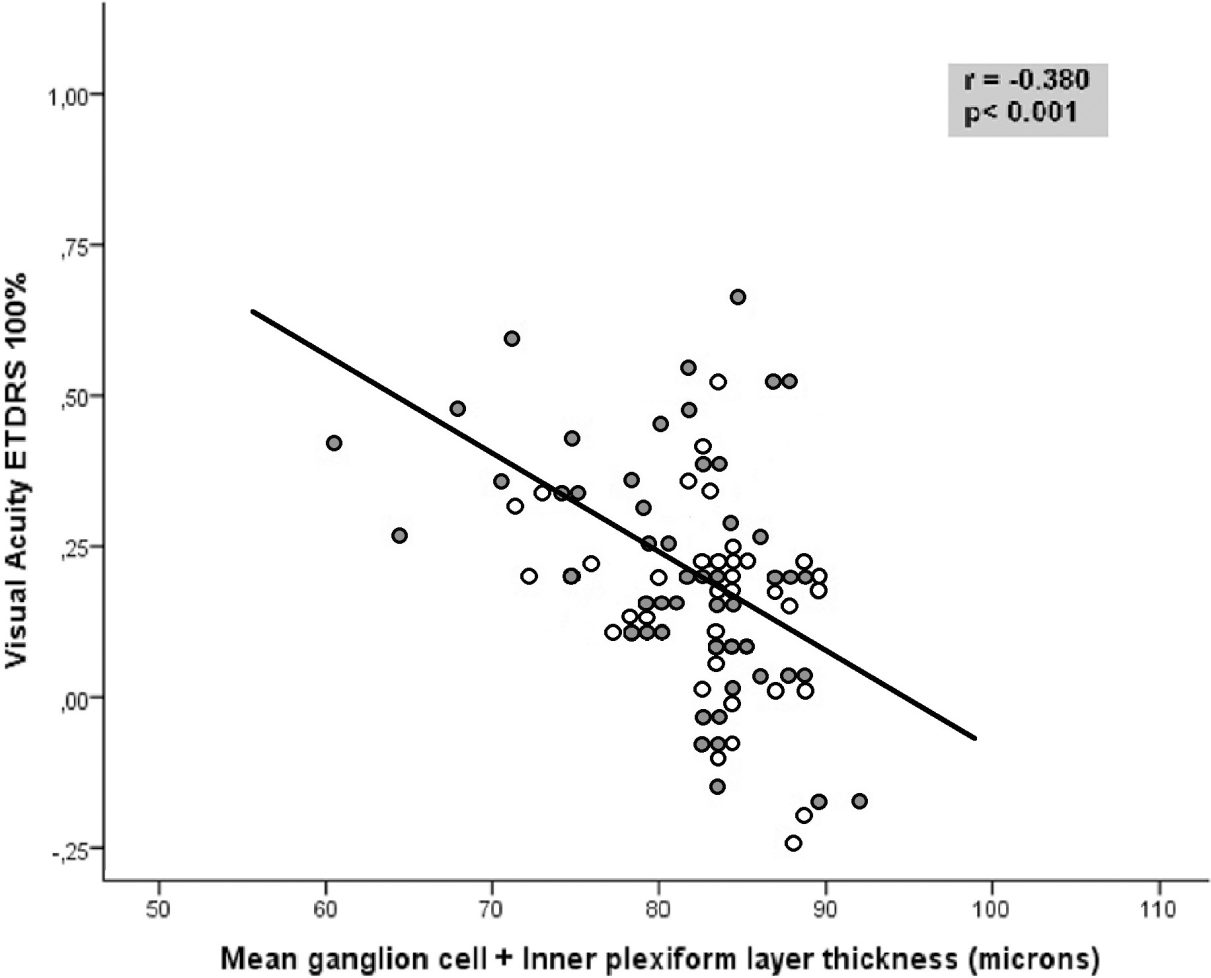
Correlation between visual acuity as measured with ETDRS optotipe at a contrast level of 100% and the average ganglion cell + inner plexiform layer thickness in patients with multiple sclerosis. Dark symbols represent data from patients with a previous episode of optic neuritis, whereas light symbols represent patients without a previous episode of optic neuritis.

VA using the ETDRS chart correlated with macular, GCIPL, and RNFL thickness (Table 4, Fig 3). There were significant but mild associations between 8 of the 9 macular parameters and LCVA at 2.50% and 1.25%, where the macular average thickness had the highest correlations (r = -0.41, p = 0.01 and r = -0.36, p = 0.04, respectively).

**Table 4:** Correlation between structural parameters and visual acuity in patients with MS.

STRUCTURAL PARAMETERS	Visual Acuity ETDRS LogMar					
	*100%*	*p*	*2*.*50%*	*p*	*1*.*25%*	*p*
**MACULAR THICKNESS**						
*Central*	-0.07	0.400	-0.07	0.430	-0.05	0.530
*Inner superior*	-0.09	0.310	-0.30	<0.001*	-0.30	0.001*
*Inner nasal*	-0.08	0.360	-0.28	0.001*	-0.26	0.002*
*Inner inferior*	-0.09	0.270	-0.32	<0.001*	-0.30	<0.001*
*Inner temporal*	-0.11	0.220	-0.25	0.003*	-0.26	0.002*
*Outer superior*	-0.06	0.460	-0.31	<0.001*	-0.32	<0.001*
*Outer nasal*	-0.04	0.670	-0.32	<0.001*	-0.29	0.001*
*Outer inferior*	-0.07	0.420	-0.26	0.002*	-0.25	0.003*
*Outer temporal*	-0.08	0.370	-0.33	<0.001*	-0.31	<0.001*
*Average*	-0.01	0.960	-0.41	0.001*	-0.36	0.004*
*Volume*	-0.04	0.720	-0.15	0.230	-0.08	0.520
**GCIPL THICKNESS**						
*Superior*	-0.42	<0.001*	-0.27	0.005*	-0.27	0.006*
*Superonasal*	-0.39	<0.001*	-0.29	0.003*	-0.28	0.005*
*Inferonasal*	-0.39	<0.001*	-0.31	0.001*	-0.28	0.004*
*Inferior*	-0.36	<0.001*	-0.26	**0.008**	-0.14	0.150
*Inferotemporal*	-0.26	**0.008**	-0.14	0.140	-0.10	0.299
*Superotemporal*	-0.32	0.001*	-0.23	**0.020**	-0.12	**0.040**
*Average GCIPL*	-0.38	<0.001*	-0.27	0.005*	-0.23	**0.020**
*Minimum GCIPL*	-0.31	0.001*	-0.21	**0.030**	-0.15	0.130
**RNFL THICKNESS**						
*Average*	-0.21	0.008*	-0.27	0.001*	-0.35	<0.001*
*Superior*	-0.21	0.010*	-0.28	<0.001*	-0.33	<0.001*
*Nasal*	-0.05	0.490	-0.04	0.630	-0.07	0.400
*Inferior*	-0.26	0.001*	-0.24	0.004*	-0.29	<0.001*
*Temporal*	-0.16	**0.040**	-0.19	**0.020**	-0.29	<0.001*

Correlation between structural parameters (macular, ganglion cell layer and retinal nerve fiber layer thickness) and visual acuity in different contrast levels in patients with multiple sclerosis. Data in bold type correspond to statistically significant correlations (p value <0.05). Asterisk indicates a significant difference after Bonferroni correction for multiple tests (p≤0.0056 for macular thickness values; p≤0.00625 for GCIPL thickness and p≤0.010 for RNFL thickness). Abbreviations: ETDRS, Early Treatment Diabetic Retinopathy Study; GCIPL, ganglion cell+inner plexiform layer; RNFL, retinal nerve fiber layer.

## Discussion

In the present study, we evaluated different structural and functional parameters and assessed the association between visual dysfunction and morphologic changes in the retina of 84 patients with MS and 84 healthy controls. We demonstrated that structural parameters, such as macular, RNFL, and GCIPL thicknesses, are reduced in MS patients, but they also exhibited visual impairment (VA and CSV reduction). Moreover, contrast sensitivity was the most affected parameter in our study and correlated with most of the structural data. We have used color vision tests that provide information for differentiating subjects with severe loss of color vision from those with milder color defects or normal color vision, and also can be used to evaluate acquired loss of color vision. Both the Farnsworth and L´Anthony tests are color arrangement tests (based on the arrangement of different color caps); however, the L´Anthony color test is less saturated, and is thus more suitable for detecting mild color anomalies that may not be detected using the Farnsworth color test alone. In our study, only the L´Anthony test results (AC CCI and Conf angle) were significantly worse in the MS group compared to healthy controls, showing a mild tendency toward protanomaly.

Visual dysfunction may occur in up to 80% of MS patients during the course of the disease.[15] Diminished contrast sensitivity and color vision deficiencies in MS have been widely reported.[16,17] Measures of low-contrast vision as tested by line gratings, letter charts, and by Pelli-Robson charts in MS patients were sensitive to visual impairment, even in patients with VA of 20/20 or better as measured with a Snellen chart. This alteration in low-contrast vision is also associated with visual impairment of everyday tasks, such as reading, driving, and facial recognition.[18] The introduction in the last few decades of OCT in the study of optic nerve neuropathies has provided new information on correlations between visual deficiencies and retinal alterations. Recent studies using OCT showed that low contrast letter acuity scores reflect the axonal and neuronal losses in the anterior visual pathways.[9,19,20] This axonal loss is also associated with disease progression, worsening disability, and lower quality of life in MS patients [21,22].

New SD-OCT segmentation software allows for the measurement of the various retinal layers. Previous studies on MS found a reduction in RNFL not only in eyes with a previous episode of ON, but also in patients who have never had an acute clinical episode of ON.[23,24] Current studies using segmentation analysis of the retinal layers have demonstrated a thinning of the GCIPL, suggesting ganglion cell loss.[9,25,26] This GCIPL thinning was significantly associated with reduced visual function and vision-specific quality of life in MS patients. Additionally GCIPL thinning occurs 3 to 6 months following acute ON.[10]

Histopathologic evaluation of postmortem MS eyes revealed the loss of inner nuclear layer neurons and significant GCIPL atrophy [27], even in cases where the number of axons remained intact.[28] Thus, GCIPL thickness has rapidly emerged as a useful structural marker in MS, even better than RNFL thickness. A positive correlation between the average GCIPL and the peripapillary RNFL thickness was recently demonstrated.[14]. Additionally, GCIPL thickness is suggested to have better sensitivity than temporal peripapillary RNFL thickness for detecting retinal thickness changes in patients with MS.[14,29]. In the present study, macular thickness was reduced in most sectors, but the peripapillary RNFL thickness was only reduced in the inferior and temporal quadrants. Segmentation analysis of the GCIPL thickness revealed a clear tendency towards a reduction in the superior and temporal (superotemporal and inferotemporal) sectors (p<0.05) although only the minimum GCIPL thickness was significantly reduced (p<0.001). These findings suggest a topographic match between defects in the GCIPL and decreased peripapillary RNFL thickness, supporting previous research, where superior macular areas (including superotemporal and superonasal sectors) anatomically correspond with the RNFL bundle to the temporal quadrant in the optic disc.[30] The thickness of these layers (especially the GCIPL) was also significantly worse in patients with a previous history of ON, corroborating previously reported results.[10, 31] Our results did not demonstrate a higher sensitivity of the GCIPL thickness to detect axonal damage. A clear tendency toward GCIPL loss in patients with MS, however, was observed (higher than in the RNFL), and comparison between groups revealed greater effects on GCIPL thickness in subjects with previous ON. Thus, further studies with a larger sample size are needed to confirm that the GCIPL reduction is a better marker of axonal damage in these patients.

Correlations between visual dysfunction and structural measurements in MS have been also reported,[9, 25, 26, 32, 33] although few of these studies include CSV and color vision analysis. A reduction in the RNFL is associated with lower LCVA, alterations in color vision, and lower quality of life in MS patients.[33, 34] The reduction of the macular and GCIPL thickness are significantly correlated with VA (high and low contrast).[9,25,26] In accordance with these previous studies, our results showed an association between macular and GCIPL thinning and worse LCVA and CSV (measured by the CSV 1000E and Pelli Robson tests), highlighting the importance of CSV tests and analysis of the GCIPL and macular thickness in the clinical evaluation of MS patients. Moreover, in our study, macular and GCIPL thicknesses were inversely correlated with L´Anthony and Farnsworth´s color indexes, respectively. These color tests evaluate the severity of dyschromatopsia and are not frequently included in studies assessing visual dysfunction and MS. In a recent study, the Farnsworth D-100 color test (based on the same principle as the Farnsworth and L´Anthony D15 color tests) was demonstrated to be more sensitive than pattern visual evoked potentials in detecting subclinical visual pathway alterations in MS patients making this color test a valuable tool for evaluating these patients.[35]

In conclusion, MS patients had reduced macular, RNFL, and GCIPL thicknesses, with the changes in the GCIPL being most closely associated with visual dysfunction. These results may be important for future investigations of neuronal and axonal loss in MS and other neurodegenerative diseases. Further studies are needed to evaluate the association between axonal injury and ganglion cell loss and to investigate the role of the GCIPL as a possible biomarker of the efficacy of neuroprotective agents.

## Supporting Information

S1 Table Mean and standard deviation (SD) of visual function parameters in healthy controls and subjects with multiple sclerosis. ANOVA test was used to compare controls and patients with history of ON and without ON. Results in bold letters indicate statistical significance (p<0.050). The brackets indicate the groups that had statistically differences in post hoc comparisons. Abbreviations: ETDRS, Early Treatment Diabetic Retinopathy Study; cpd, cycles per degree; AC CCI, age-corrected color confusion index; Conf Angle, confusion angle; S-index, scatter index; MS, multiple sclerosis; ON, optic neuritis; C, controls.
(DOCX)

S2 Table Mean and standard deviation (SD) of structural parameters in healthy controls and subjects with multiple sclerosis. ANOVA test was used to compare controls and patients with history of ON and without ON (no-ON). Results in bold letters indicate statistical significance (p<0.050). The brackets indicate the groups that had statistical differences in post hoc comparisons. Abbreviations: GCIPL, Ganglion cell + inner plexiform layer; RNFL, retinal nerve fiber layer; MS, multiple sclerosis; ON, optic neuritis; C, controls.
(DOCX)
