# Supplementary material for: Relationship between Visual Dysfunction and Retinal Changes in Patients with Multiple Sclerosis
Source: PLoS One. 2016 Jun 28;11(6):e0157293. doi: 10.1371/journal.pone.0157293 (PMC4924797; doi:10.1371/journal.pone.0157293)
Supplement: S1 Table — ANOVA test was used to compare controls and patients with history of ON and without ON. Results in bold letters indicate statistical significance (p<0.050). The brackets indicate the groups that had statistically differences in post hoc comparisons. Abbreviations: ETDRS, Early Treatment Diabetic Retinopathy Study; cpd, cycles per degree; AC CCI, age-corrected color confusion index; Conf Angle, confusion angle; S-index, scatter index; MS, multiple sclerosis; ON, optic neuritis; C, controls. (DOCX) [file pone.0157293.s001.docx]

|  | | **CONTROL** | **MS no ON** | **MS with ON** | **P** | **POST HOC** |
| --- | --- | --- | --- | --- | --- | --- |
| **FUNCTIONAL EXAMINATION** | **VISUAL ACUITY** |  |  |  |  |  |
|  | *ETDRS 100* | -0.09 (0.09) | 0.07 (0.20) | 0.20 (0.94) | **0.004** | **[C:ON]** |
|  | *ETDRS 2.5* | 0.42 (0.12) | 0.53 (0.15) | 0.58 (0.19) | **<0.001** | **[C:no-ON] [C:ON]** |
|  | *ETDRS 1.25* | 0.55 (0.14) | 0.70 (0.15) | 0.72 (0.17) | **<0.001** | **[C:no-ON] [C:ON]** |
|  | **CONTRAST SENSITIVITY** |  |  |  |  |  |
|  | *Pelli Robson* | 1.89 (0.11) | 1.77 (0.23) | 1.69 (0.23) | **<0.001** | **[C:no-ON] [C:ON]** |
|  | *CSV 1000 3 cpd* | 1.73 (0.19) | 1.45 (0.20) | 1.43 (0.14) | **<0.001** | **[C:no-ON] [C:ON]** |
|  | *CSV 1000 6 cpd* | 1.98 (0.19) | 1.65 (0.22) | 1.40 (0.15) | **0.004** | **[C:no-ON] [C:ON]** |
|  | *CSV 1000 12 cpd* | 1.62 (0.22) | 1.01 (0.23) | 0.99 (0.29) | **<0.001** | **[C:no-ON] [C:ON]** |
|  | *CSV 1000 18 cpd* | 1.16 (0.21) | 0.69 (0.14) | 0.50 (0.18) | **0.023** | **[C:no-ON] [C:ON]** |
|  | **CHROMATIC VISION** |  |  |  |  |  |
|  | *CVR Farnsw AC CCI* | 1.11 (0.43) | 1.12 (0.27) | 4.31 (1.73) | 0.206 |  |
|  | *CVR Farnsw ConfAngle* | 56.08 (7.93) | 63.72 (7.78) | 55.80 (6.78) | 0.463 |  |
|  | *CVR Farnsw S- Index* | 1.66 (0.43) | 1.75 (0.56) | 1.73 (0.43) | 0.603 |  |
|  | *CVR L’Anthony AC CCI* | 1.13 (0.36) | 1.28 (0.37) | 1.25 (0.25) | 0.098 |  |
|  | *CVR L’Anthony ConfAngle* | 55.49 (4.02) | 41.30 (9.91) | 39.51 (6.58) | 0.141 |  |
|  | *CVR L’Anthony S-Index* | 1.79 (0.50) | 1.74 (0.42) | 1.86 (0.40) | 0.629 |  |

**Supplementary table 1:** Mean and standard deviation (SD) of visual function parameters in healthy controls and subjects with multiple sclerosis. ANOVA test was used to compare controls and patients with history of ON and without ON. Results in bold letters indicate statistical significance (p<0.050). The brackets indicate the groups that had statistically differences in post hoc comparisons. Abbreviations: ETDRS, Early Treatment Diabetic Retinopathy Study; cpd, cycles per degree; AC CCI, age-corrected color confusion index; Conf Angle, confusion angle; S-index, scatter index; MS, multiple sclerosis; ON, optic neuritis; C, controls.
