# Supplementary material for: Relationship between Visual Dysfunction and Retinal Changes in Patients with Multiple Sclerosis
Source: PLoS One. 2016 Jun 28;11(6):e0157293. doi: 10.1371/journal.pone.0157293 (PMC4924797; doi:10.1371/journal.pone.0157293)
Supplement: S2 Table — ANOVA test was used to compare controls and patients with history of ON and without ON (no-ON). Results in bold letters indicate statistical significance (p<0.050). The brackets indicate the groups that had statistical differences in post hoc comparisons. Abbreviations: GCIPL, Ganglion cell + inner plexiform layer; RNFL, retinal nerve fiber layer; MS, multiple sclerosis; ON, optic neuritis; C, controls. (DOCX) [file pone.0157293.s002.docx]

|  | | **CONTROL** | **MS no ON** | **MS with ON** | **P** | **POST HOC** |
| --- | --- | --- | --- | --- | --- | --- |
| **STRUCTURAL EXAMINATION** | **MACULAR THICKNESS** |  |  |  |  |  |
|  | *Fovea* | 257.07 (16.33) | 252.38 (14.67) | 246.23 (13.26) | **0.005** | **[C:no-ON] [C:ON] [no-ON:ON]** |
|  | *Inner superior sector* | 325.99 (13.37) | 311.53 (26.22) | 308.37 (19.13) | **<0.001** | **[C:no-ON] [C:ON]** |
|  | *Inner nasal sector* | 326.35 (13.56) | 311.71(28.38) | 307.74 (19.94) | **<0.001** | **[C:no-ON] [C:ON]** |
|  | *Inner inferior sector* | 321.96 (14.00) | 305.76 (27.17) | 303.26 (20.40) | **<0.001** | **[C:no-ON] [C:ON]** |
|  | *Inner temporal sector* | 311.14 (12.24) | 301.41 (23.67) | 294.32 (19.82) | **<0.001** | **[C:no-ON] [C:ON]** |
|  | *Outer superior sector* | 283.21 (11.66) | 273.41 (20.54) | 273.47 (18.21) | **0.006** | **[C:no-ON] [C:ON]** |
|  | *Outer nasal sector* | 299.80 (14.97) | 286.81(24.99) | 278.05 (22.24) | **<0.001** | **[C:no-ON] [C:ON]** |
|  | *Outer inferior sector* | 272.04 (13.02) | 266.76 (29.16) | 257.95 (15.77) | **0.019** | **[C:ON]** |
|  | *Outer temporal sector* | 264.79 (11.86) | 257.47 (15.24) | 252.16 (13.24) | **<0.001** | **[C:ON]** |
|  | *Average* | 291.55 (15.70) | 279.60 (16.01) | 261.04 (14.44) | **<0.001** | **[C:no-ON] [C:ON] [no-ON:ON]** |
|  | *Volume* | 10.12 (0.54) | 9.95 (0.54) | 9.77 (0.50) | **<0.001** | **[C:no-ON [C:ON] [no-ON:ON]** |
|  | **GCIPL THICKNESS** |  |  |  |  |  |
|  | *Superior sector* | 85.58 (6.67) | 80.42 (5.88) | 71.00 (8.29) | **<0.001** | **[C:ON] [C:no-ON] [no-ON:ON]** |
|  | *Superonasal sector* | 85.85 (7.46) | 80.08 (7.96) | 71.27 (8.60) | **<0.001** | **[C:ON] [C:no-ON] [no-ON:ON]** |
|  | *Inferonasal sector* | 84.61 (7.55) | 79.83 (8.54) | 70.27 (8.32) | **<0.001** | **[C:ON] [no-ON:ON]** |
|  | *Inferior sector* | 83.59 (7.70) | 80.17 (5.96) | 67.91 (8.71) | **<0.001** | **[C:ON] [no-ON:ON]** |
|  | *Inferotemporal sector* | 84.17 (6.73) | 80.83 (3.46) | 66.36 (15.66) | **<0.001** | **[C:ON] [no-ON:ON]** |
|  | *Superotemporal sector* | 83.87 (6.23) | 79.17 (6.80) | 67.00 (10.78) | **<0.001** | **[C:ON] [no-ON:ON]** |
|  | *Average GCIPL* | 84.68 (6.74) | 80.25 (5.29) | 69.00 (7.55) | **<0.001** | **[C:ON] [no-ON:ON]** |
|  | *Min GCIPL* | 82.42 (6.50) | 76.67 (7.31) | 63.09 (16.02) | **<0.001** | **[C:ON] [C:no-ON] [no-ON:ON]** |
|  | **RNFL THICKNESS** |  |  |  |  |  |
|  | *Average* | 94.35 (9.62) | 87.47 (10.94) | 81.21 (14.64) | **<0.001** | **[C:ON]** |
|  | *Superior sector* | 117.10 (17.20) | 110.42(17.17) | 103.74 (18.06) | **0.010** | **[C:ON]** |
|  | *Nasal sector* | 69.93 (11.79) | 68.63 (12.90) | 66.37 (15.42) | 0.547 |  |
|  | *Inferior sector* | 124.1 (14.81) | 109.58(13.50) | 105.68 (22.99) | **<0.001** | **[C:no-ON] [C:ON] [no-ON:ON]** |
|  | *Temporal sector* | 64.14 (8.94) | 61.79 (21.03) | 49.21 (11.99) | **<0.001** | **[C:ON] [no-ON:ON]** |

**Supplementary table 2:** Mean and standard deviation (SD) of structural parameters in healthy controls and subjects with multiple sclerosis. ANOVA test was used to compare controls and patients with history of ON and without ON (no-ON). Results in bold letters indicate statistical significance (p<0.050). The brackets indicate the groups that had statistical differences in post hoc comparisons. Abbreviations: GCIPL, Ganglion cell + inner plexiform layer; RNFL, retinal nerve fiber layer; MS, multiple sclerosis; ON, optic neuritis; C, controls.
